# Fast-switching laterally virtual-moving microlens array for enhancing spatial resolution in light-field imaging system without degradation of angular sampling resolution

**DOI:** 10.1038/s41598-019-47819-9

**Published:** 2019-08-05

**Authors:** Min-Kyu Park, Heewon Park, Kyung-Il Joo, Tae-Hyun Lee, Ki-Chul Kwon, Munkh-Uchral Erdenebat, Young-Tae Lim, Nam Kim, Hak-Rin Kim

**Affiliations:** 1 School of Electronics Engineering, Kyungpook National University, 80 Daehak-ro, Buk-gu, Daegu 41566 South Korea; 2 School of Information and Communication Engineering, Chungbuk National University, 1 Chungdae-ro, Seowon-gu, Cheongju, Chungbuk 28644 South Korea

**Keywords:** Electrical and electronic engineering, Applied optics, Imaging and sensing

## Abstract

We present an electrically controllable fast-switching virtual-moving microlens array (MLA) consisting of a stacked structure of two polarization-dependent microlens arrays (PDMLAs) with optical orthogonality, where the position of the two stacked PDMLAs is shifted by half the elemental pitch in the diagonal direction. By controlling the polarization of the incident light without the physical movement of the molecules comprising the virtual-moving MLA, the periodic sampling position of the MLA can be switched fast using a polarization-switching layer based on a fast-switching liquid crystal cell. Using the fast-switching virtual-moving MLA, the spatial-resolution-enhanced light-field (LF) imaging system was demonstrated without a decrease in the angular sampling resolution as compared to the conventional LF imaging system comprising a passive MLA; two sets of elemental image arrays were captured quickly owing to the short switching time of the virtual-moving MLA of 450 μs. From the two captured sets of the elemental array image, four-times resolution-enhanced reconstruction images of the directional-view and depth-slice images could be obtained.

## Introduction

Since Gabor proposed the use of an array of microscale lenses as a superlens to overcome the diffraction limit^1^, microlens arrays (MLAs) have been widely used in optical systems for imaging or non-imaging applications including charge-coupled device (CCD) cameras^2–4^, light-field (LF) imaging systems^5–8^ or LF 3D displays^9–11^, sensors^12,13^, photolithography^14^, fibre couplers^15^, optical switches^16,17^, and light-emitting diodes (LEDs) or organic-LEDs (OLEDs)^18,19^. In particular, MLAs in LF imaging systems are used to capture optical information on directional ray distributions. Based on the 3D ray vector information sampled by the MLA, the full-parallax images or depth-slice images can be reconstructed from the captured elemental image array^5–8^. However, current LF imaging schemes have a severe problem of low imaging resolutions to be implemented practical optical systems that is too low than those of conventional 2D imaging systems. To improve the resolution of the reconstructed images in the LF imaging system, the density of the elemental lens of the LF imaging system should be increased with a high fill factor. Various fabrication methods have been introduced for obtaining an MLA with a higher fill factor: these include lithography^20^, laser writing^21^, wet etching^22^, hot-embossing process^23^, photoresist reflow^24^, and inkjet printing^25^. However, with an increase in the elemental lens density owing to the use of these passive types of MLAs, under the condition of an image sensor with a fixed pixel density, the angular sampling resolution of the LF imaging system is inevitably degraded because of the decreased ray direction sampling under the condition of a reduced elemental lens aperture.

Some recent studies have been focused on improving the spatial resolution of the LF imaging system without causing a degradation in the angular sampling resolution by introducing laterally shifting schemes of MLAs. A resolution-enhanced LF recoding and display system that uses synchronously movable MLA was presented by J.-S. Jang *et al*.^10^. Y.-T. Lim *et al*. presented resolution-enhanced LF imaging microscopy that comprised the use of a movable MLA controlled by a piezo-electric actuator^26^. However, these types of mechanically moving MLAs are difficult to implement with a compact optical module and make it difficult to control the sampling synchronization precisely.

Liquid crystals (LCs) are electro-optic (EO) materials that can be used to modulate the relative phase profiles of incident rays depending on the field-induced molecular distribution of the LCs. Using the EO properties of LCs, the gradient refractive index (GRIN) LC profiles can be electrically tailored as a switchable lens. T.-H. Jen *et al*. presented an electrically controllable moving MLA based on periodic LC GRIN profiles, which are controlled using fringe electric-field distributions generated by patterned indium-tin-oxide electrodes^27^. In this structure, two electrically movable lenticular LC lens array devices were orthogonally stacked to obtain a 2D lens array. However, the fill factor of the MLA is insufficient for implementation in the time-multiplexed ray sampling of resolution-enhancing LF imaging systems because the LC molecules on the digitized electrode patterns are not effectively controlled by the fringe fields^28,29^. To obtain a wider field-of-view imaging condition, an MLA having a shorter focal length (or a smaller *f*-number) is required; however, this results in an LC-based GRIN lens design with quite a thick LC layer thickness. This inevitably results in an increase in its operating voltage and considerably slows down the switching dynamics (~2 s^27^) to be used as high-speed image capturing with the time-multiplexing schemes.

In this paper, we present a fast, electrical switching MLA scheme exhibiting laterally shifting periodic-imaging samplings with a high fill factor that can generate virtually moving optical effects of the periodically focusing MLA without any physically moving part. The virtual-moving MLA consists of a stacked MLA structure, wherein each MLA has two polarization-dependent focusing properties and diagonally shifted periodic image samplings. In this virtual-moving MLA, the periodic ray-sampling positions of each elemental lens array can be shifted quite fast according to an incident polarization state controlled by a polarization-switching layer. By using the orthogonality of the optical polarizations with the virtual-moving MLA, two sets of the elemental image arrays can be sequentially captured in the field-on and field-off response times of 270 μs and 180 μs, respectively, using the time-multiplexing spatial sampling scheme. The resolution enhancement achieved with the LF imaging system on using the virtual-moving MLA is discussed for the reconstruction images of the directional views and the depth-slice refocused images.

## Fast-Switching Virtual-Moving MLA

**Figure 1:**
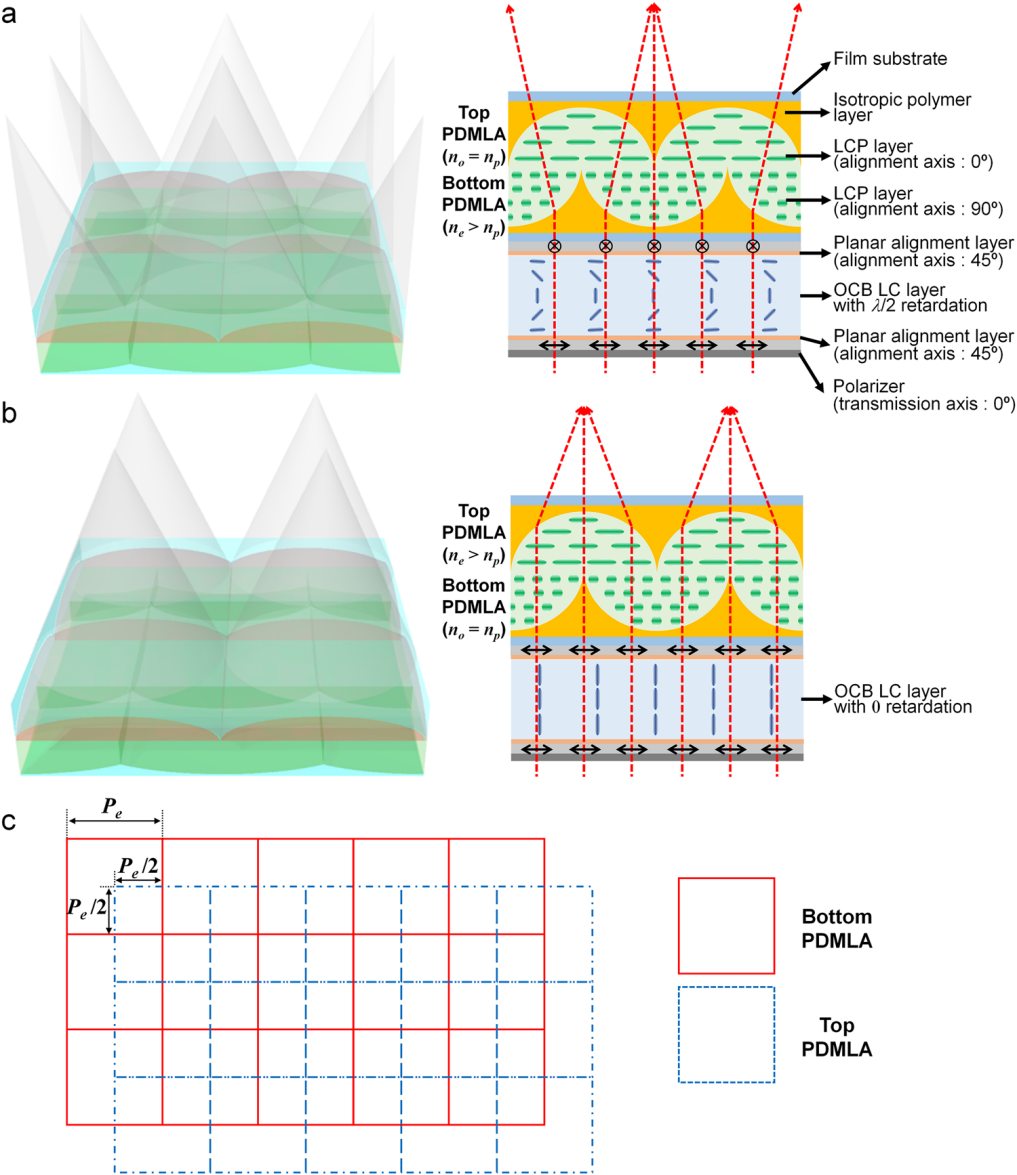
Device structure and operating principle of the virtual-moving MLA. (**a**,**b**) Laterally shifted periodic beam-focusing states obtained by the bottom and top polarization-dependent MLA (PDMLA) according to the applied field conditions of the polarization-switching optically compensated bend (OCB) LC layer. (**c**) Top-view schematic of the virtual-moving MLA, wherein the lateral displacement of the bottom and top PDMLA arrangement is set as half the pitch of the elemental lens array in the diagonal direction.

For enhancing the spatial resolution of the LF imaging without causing a degradation in the angular sampling resolution, the approach of the lens shifting method, the virtual-moving MLA is implemented to obtain the time-sequential elemental image acquisition as shown in Fig. 1. The virtual-moving MLA is obtained through the integration of two polarization-dependent MLAs (PDMLAs), and the elemental lens position of the two PDMLAs are relatively shifted by half the pitch of the elemental lens array. Each PDMLA consists of a birefringent liquid crystalline polymer (LCP) layer having a planar-convex lens shape and an isotropic polymer layer having a concave-planar shape, wherein the ordinary refractive index (*n*_*o*_) of the LCP layer is the same as the refractive index (*n*_*p*_) of the isotropic polymer layer, and the extra-ordinary refractive index (*n*_*e*_) of the LCP layer is larger than *n*_*p*_. The *n*_*e*_ axes of the LCP layer of the top and bottom PDMLAs are aligned orthogonally with each other, i.e., along the *x*-axis direction (0°) and *y*-axis direction (90°), respectively. In this structure, in the case of the *y*-axis polarization state, the incident rays propagate through the top PDMLA without an optical refraction owing to the index-matching condition between the LCP layer and the isotropic polymer layer, and they are periodically focused in propagating through the bottom PDMLA, as shown in Fig. 1a. In contrast, when the incident polarization state is switched to the *x*-axis direction, the periodic ray focusing is obtained during propagation through the top PDMLA as shown in Fig. 1b. Figure 1c shows the top-view schematic of the relative elemental lens arrangement of the stacked PDMLAs. The position of the top PDMLA is shifted along the diagonal direction by half the pitch of the elemental lens array as compared to that of the bottom PDMLA.

**Figure 2:**
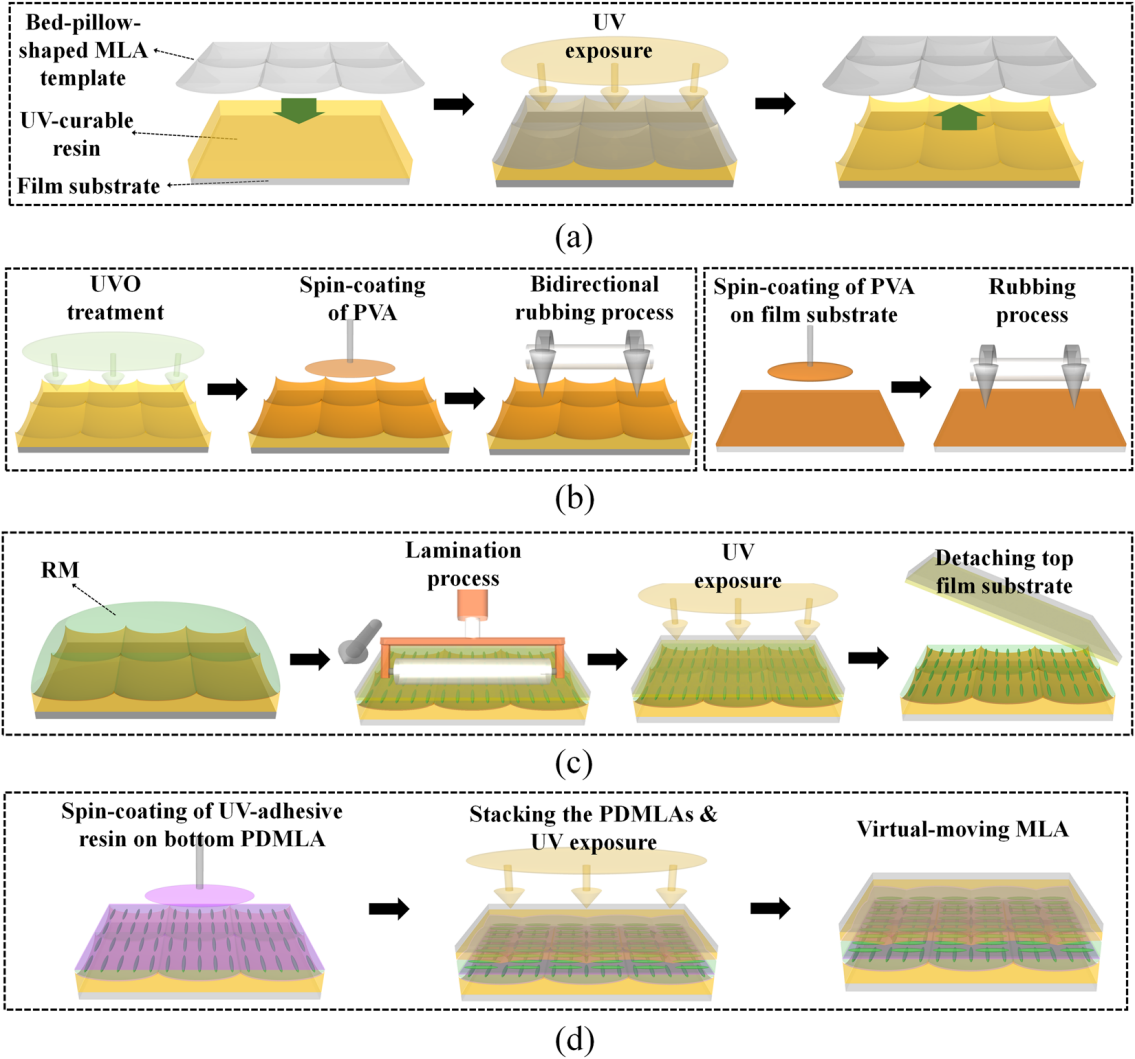
Schematics of the fabrication procedures of the virtual-moving MLA: (**a**) ultraviolet (UV) nano-imprinting process for fabricating the planar–concave MLA structure made of the optically isotropic UV-curable resin, (**b**) UVO surface treatment on the replica-moulded planar–concave MLA structure, spin-coating and curing of the alignment layer (PVA), bi-directional rubbing treatment, and preparation of the top–down alignment film by spin-coating and curing of the PVA layer and rubbing treatment. (**c**) One-drop filling process of the reactive mesogen (RM) solution, lamination of the top–down alignment film, UV curing for the polymerization of the RM layer, and peel-off procedure of the top–down alignment film. (**d**) Lamination of the top PDMLA on the bottom PDMLA using the UV curable adhesive material between two MLAs, and the final structure of the virtual-moving MLA.

**Figure 3:**
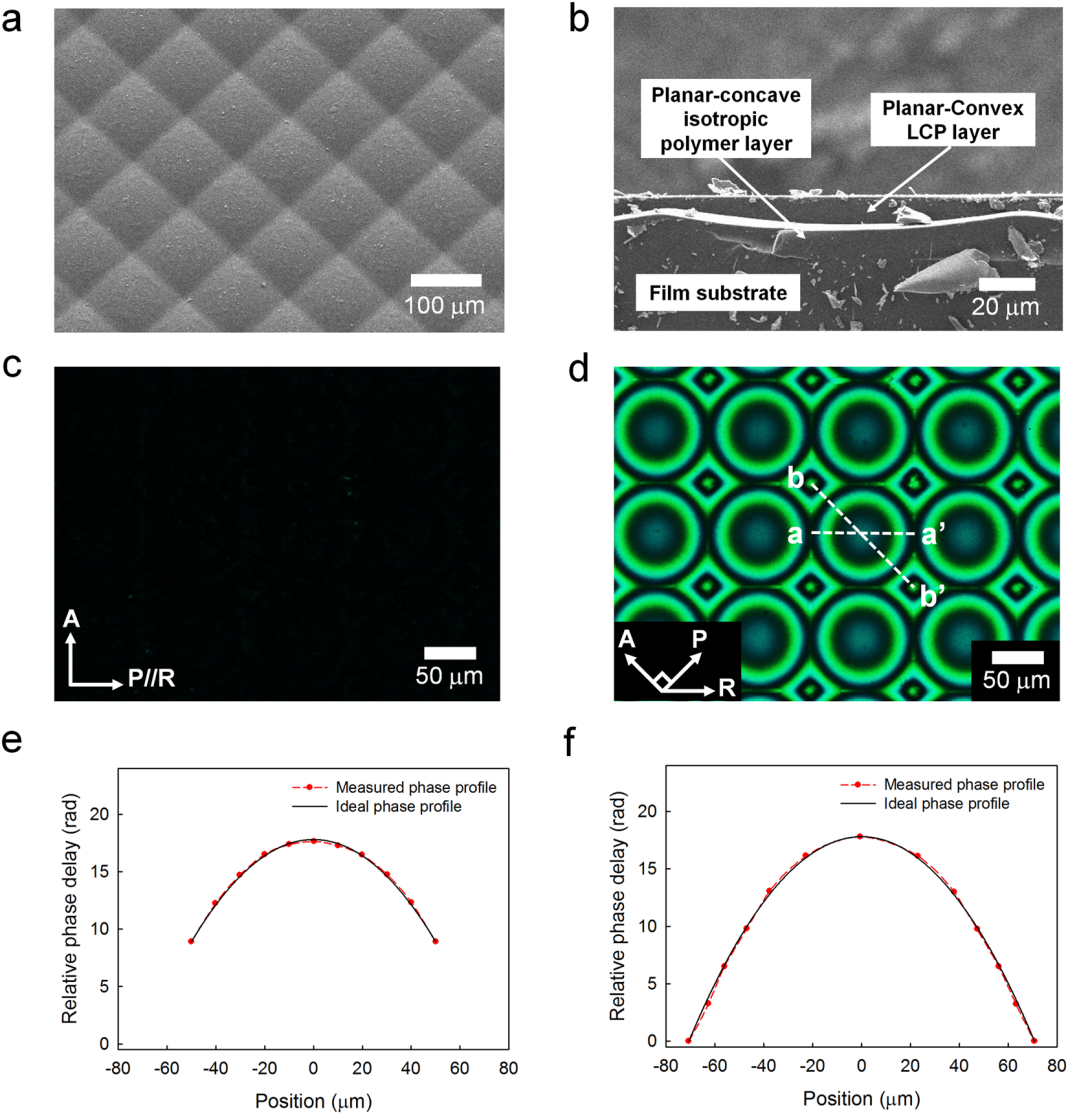
(**a**) Oblique view of the scanning electron microscope (SEM) image of the MLA with the square-arranged bed-pillow-shaped elemental lens surface topology, which is used as the master mould during the nano-imprinting process. (**b**) Cross-sectional view of the SEM image of the single PDMLA. (**c**,**d**) Polarizing optical microscope images of the single PDMLA observed between the crossed polarizers, wherein the rubbing direction is (**c**) parallel and (**d**) at 45° with respect to the transmission axis of one of the polarizers. (**e**,**f**) Measured and ideal relative phase delay profiles of the single PDMLA, wherein the measured results are derived from the phase unwrapping of the fringe ring patterns shown in (**d**) along the sampling directions of (**e**) a–a’ and (**f**) b–b’.

Figure 2 shows the fabrication process of each PDMLA layer and the procedure of its stacking for fabricating the virtual-moving MLA. First, for each PDMLA, by performing a UV-nanoimprinting process, the isotropic polymer layer with the concave-planar MLA shape was formed using a UV-curable polymer resin of NOA89 (Norland Products Inc.; *n*_*p*_ = 1.51) as shown in Fig. 2a. In this experiment, the square-arranged bed-pillow-shaped MLA template (MLA-S100-f4, RPC Photonics) was used for the preparation of the soft mould template required for the UV-nanoimprinting process. The oblique view of the surface profile of the MLA template is shown in Fig. 3a with the scanning electron microscope (SEM) image. With the periodic surface topology shown in Fig. 3a, the PDMLA with a fill factor of nearly 100% can be achieved, wherein the pitch and height of the square-shaped elemental lens is 100 μm and 5.3 μm, respectively. After the UV-nanoimprinting process, in order to obtain the anisotropic optical properties of the LCP molecules on the planar–concave isotropic MLA, we applied bottom–up and top–down interfacial alignment effects with a rubbing process on the bottom planar–concave MLA and on the other upper flat film surface, which are shown in Fig. 2b,c. The surface of the planar–concave MLA structure and the upper flat film were treated using a UV ozone process for 30 min in order to create a hydrophilic surface for the post-coating process. Polyvinyl alcohol (PVA) dissolved in water was coated onto the planar–concave MLA structure and the upper flat film, and they were thermally annealed at 90 °C for 30 min. The PVA layers were then bi-directionally rubbed to avoid shading regions on the planar–concave MLA surface. For the LCP layer, we used a UV-curable reactive mesogen (RM) (RMM727, Δ*n* = 0.19, Merck). The LCP layer was formed by casting the LC RM on the planar–concave MLA structure, laminating the upper film substrate onto it, and polymerizing the RM using UV irradiation (for 90 s at 50 mW/cm^2^) in a uniaxially aligned state. Subsequent to the UV-induced polymerization of the RM LCP layer, the upper film substrate applied for the top–down LC alignment was peeled off, as shown in Fig. 2c. For the virtual-moving MLA, the other set of the PDMLA was prepared using the same method described above; however, the rubbing direction used was orthogonal to each other. The two PDMLAs with the orthogonal slow axis of the LCP layer were laminated with each other using an optically clear resin (NOA1625, Norland Products Inc.; *n* = 1.625), as shown in Fig. 2d, wherein the elemental lens positions of the two PDMLAs were aligned to be shifted by half the pitch of the elemental lens along the diagonal direction utilizing a micro-moving stage with a microscope. As all the optical layers consisting of the stacked PDMLAs were UV-curable polymeric layers on the film substrates, the printing process is viable without inflicting chemical, thermal, or mechanical stress damages being inflicted on the flexible film substrates.

Figure 3b shows the cross-sectional SEM image of one of the fabricated PDMLAs before it was laminated. The total thickness of a single PDMLA layer was approximately 120 μm including the film substrate (100 μm), where the residual thickness of the LCP layer on the film substrate was approximately 3.5 μm. The optically clear resin layer used for laminating the two PDMLAs with each other was approximately 20 μm thick, and the total thickness of the stacked structure as the virtual-moving MLA was approximately 260 μm including the two film substrates—this stacked structure was thus thin and flexible. As shown in Fig. 2d, two polymerized PDMLA films could be stacked with a very thin adhesion layer without additional guiding substrates between them in contrast to the case of an LC-based switchable lens, and the longitudinal deviation of the focal planes between the top and bottom PDMLAs could be minimized for the time-multiplexed LF imaging.

Figure 3c,d show the polarized optical microscope (POM) images of the PDMLA observed through the crossed polarizers. As the slow axis of the LCP layer of the PDMLA was set such that it was parallel to the transmission axis of one of the polarizers, a completely dark LCP texture could be obtained without any optical leakage observation, as shown in Fig. 3c. In this figure, the RM molecules were well aligned along the alignment direction without an optic axis deviation due to the bottom–up and top–down interfacial alignment effects, although there were the periodic square-arranged planar–concave topology as the LCP alignment bottom surface. As the slow axis of the LCP layer of the PDMLA was rotated to 45° with respect to the transmission axes of the crossed polarizers, bright and dark ring patterns could be observed owing to the positional retardation variation of the planar–convex LCP layer, as shown in Fig. 3d, wherein a green filter was inserted into the POM for observing clear fringe patterns. The results show that all the phase profiles of each elemental lens were ideally centro-symmetric, and a fill factor of the PDMLA of nearly 100% could be achieved using the square-shaped bed-pillow-shaped MLA structure. Using the bright and dark ring patterns and the positional relative light transmittance levels shown in Fig. 3d, the effective positional relative retardation within an elemental lens was derived after the phase unwrapping. Figure 3e,f show the relative phase delay of the PDMLA for one region of the elemental lens array along the horizontal (a–a’) and diagonal (b–b’) directions shown in Fig. 3d. The focal length of the PDMLA can be estimated using the following equation^30,31^:

1 $$f=\pi {r}^{2}/\phi \lambda ,$$

where *r* is the aperture radius of each elemental lens, *φ* is the maximum phase difference between the centre and edge positions of the elemental lens, and *λ* is the wavelength of the incident light (*λ* = 550 nm). The derived focal length and *f*-number obtained using Eq. (1) were 1.60 mm and *f*/16, respectively.

**Figure 4:**
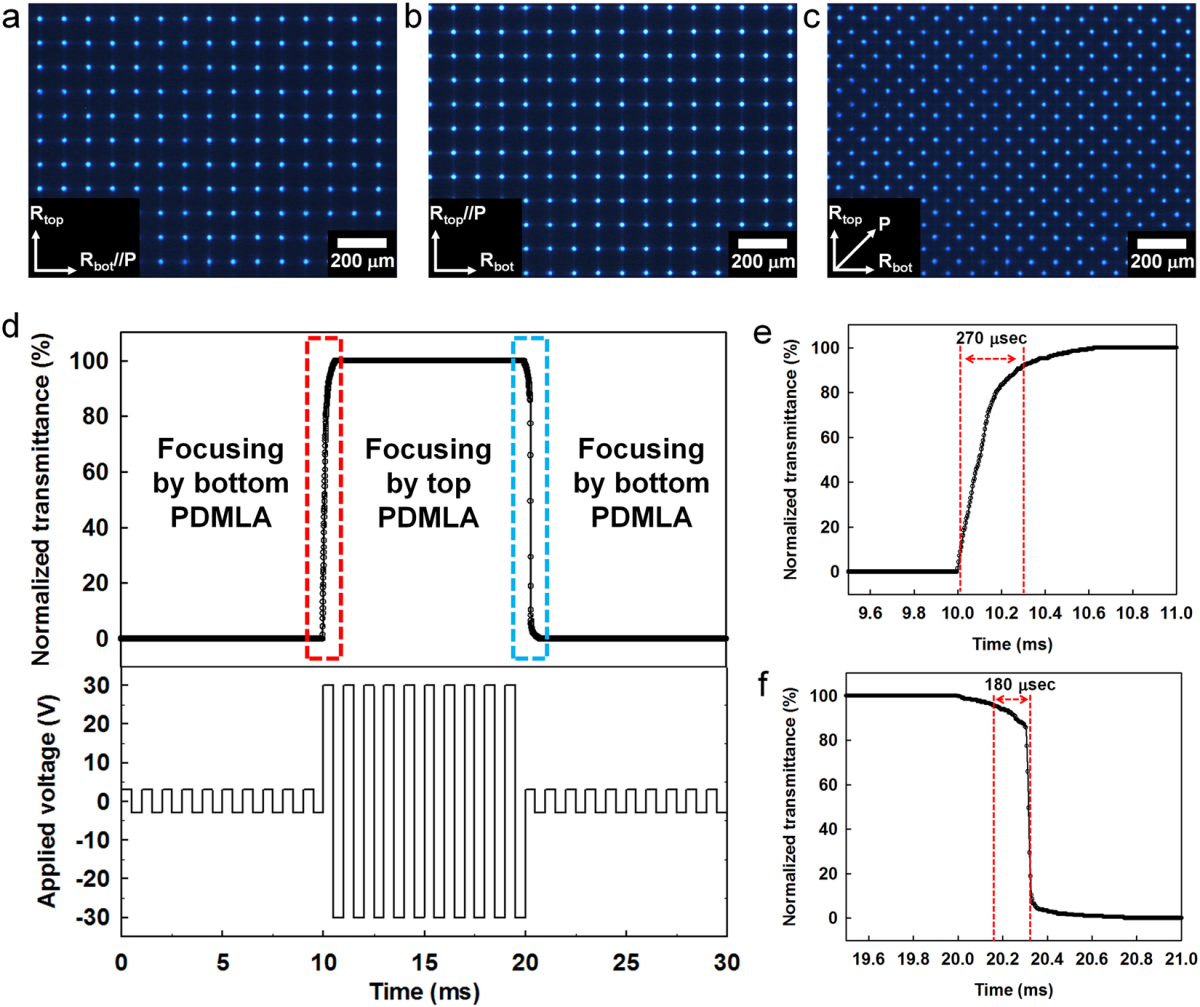
(**a**–**c**) Optical microscope images of the focused beam patterns obtained using the virtual-moving MLA that are captured at the focal plane when the incident polarization states are parallel with the rubbing direction for the RM layer of (**a**) the top PDMLA and (**b**) bottom PDMLA and (**c**) rotated by 45° with respect to both the rubbing directions. (**d**) Time-dependent switching characteristics of the polarization switching layer (OCB mode LC cell) according to the applied field conditions. (**e**,**f**) Enlarged graphs of (**d**) presented to characterize the switching dynamics of the virtual-moving MLA. For the moving picture showing the fast-switching laterally moved focusing properties, see Movie S1.

Figure 4a–c show the focused beam patterns of the virtual-moving MLA according to the polarization states of the incident cool white beam. When the polarization state of the incident beam is parallel to the slow axis of the bottom PDMLA, the periodic focused beam pattern is determined by the bottom PDMLA, as shown in Figs 1a and 4a. In contrast, when the incident polarization is parallel to the slow axis of the top PDMLA, the periodic focused beam pattern is determined by the top PDMLA, as shown in Figs 1b and 4b. Figure 4c shows the focusing behaviour obtained under the incident polarization condition of 45° with respect to both alignment directions of the LCP layers of the top and bottom PDMLAs, which exhibits both beam patterns focused by the top and bottom PDMLAs. In Fig. 4c, as the lens planes can be considered to have approximately the same longitudinal position in our stacked PDMLAs, the beam profiles focused by the top and bottom PDMLAs were almost identical at the same focal plane.

Considering the full-colour LF imaging utilizing the virtual-moving MLA scheme, two dispersion characteristics of the *n*_*o*_ values of the planar-convex LCP layer and the *n*_*p*_ values of the planar-concave isotropic polymer layer should be matched each other over the whole visible range to avoid the optical crosstalk between two sets of the laterally shifting LF image acquisitions by the time-sequentially operating stacked PDMLAs. When we measured the refractive indices of *n*_*o*_ and *n*_*e*_ for the LCP and the *n*_*p*_ refractive index for the isotropic polymer with an interferometric method, the wavelength-dependent refractive indices are *n*_*o*_ = 1.5592, *n*_*e*_ = 1.7511, and *n*_*p*_ = 1.5652 at *λ* = 450 nm (blue), and *n*_*o*_ = 1.5136, *n*_*e*_ = 1.6794, and *n*_*p*_ = 1.5111 at *λ* = 550 nm (green), and *n*_*o*_ = 1.4952, *n*_*e*_ = 1.6508, and *n*_*p*_ = 1.4892 at *λ* = 650 nm (red). From the measured values, we found the dispersion equations of *n*_*o*_(*λ*), *n*_*e*_(*λ*), and *n*_*p*_(*λ*) by curve-fitting using the Cauchy equation (See Supplementary Section 1). Two dispersion curves of *n*_*o*_(*λ*) and *n*_*p*_(*λ*) showed that the index matching condition between the planar-convex LCP and planar-concave isotropic polymer layers of the stacked structure of the PDMLAs were preserved well over the whole visible range for the ordinary incident rays. The average value of the index mismatching amount over the visible range (400 nm~700 nm) was extremely low with $< |{n}_{o}-{n}_{p}| >$ = 0.005615. The experimental results of the polarization-dependent focused beam patterns shown in Fig. 4a,b also show ideal switching characteristics without a crosstalk in the virtual-moving focused beam patterns although the incident beam condition for Fig. 4a,b were the broadband cool white visible source. For the operation of the beam-focused state of the PDMLA using the incident polarization condition of the extraordinary ray, the dispersion curve of *n*_*e*_(*λ*) is different with *n*_*p*_(*λ*) unlike the refractive index relationship between *n*_*o*_(*λ*) and *n*_*p*_(*λ*) because of the more dispersive characteristics of *n*_*e*_(*λ*) than *n*_*o*_(*λ*) of the uniaxial molecular structure of the LCP. This caused the chromatic aberration at the focused state of the PDMLA. In our experiment, to acquire the full colour LF imaging, the depth of field condition of the PDMLA was designed to be sufficiently large by adopting the large *f*-number (*f*/16) elemental lens condition. The details on the characterization methods and their results of the material dispersions (Fig. S1a), the index matching properties (Fig. S1a,b) between *n*_*o*_(*λ*) and *n*_*p*_(*λ*) for the polarization-dependent switching function in the stacked structure, and the chromatic aberration properties with the wavelength-dependent focusing (Fig. S1c) and wavelength-dependent imaging evaluated by the modulation transfer function (Fig. S1d) are presented in Supplementary Information (See Supplementary Section 1).

In our resolution-enhanced LF imaging system that uses the time-multiplexing scheme with the virtual-moving MLA, the time-sequential periodic ray sampling by each PDMLA layer is switched by the incident polarization change of the underlying polarization switching layer made by the fast-switching LC cell. Thus, the fast-switching property for the incident polarization control is important, and an optically compensated bend (OCB) LC mode cell is used as the polarization switching layer in our implementation of the LF imaging system. The OCB mode can provide an extremely short field-off response time as compared to those of the other types of LC modes owing to the field-off initial LC geometry of the large band elastic deformation. Figure 4d–f illustrate the switching dynamics between the two orthogonal output polarization states of the polarization switching layer required for two sets of the periodic ray sampling performed using the virtual-moving MLA. In Fig. 4d, the field waveform applied to the OCB mode LC cell used as the polarization switching layer was co-plotted. For the focusing states obtained by the bottom and top PDMLAs, 30 V_p_ and 3 V_p_ at 1 kHz, respectively, were applied to the polarization switching layer, wherein the polarization switching LC layer had a zero and half wave phase retardation for *λ* = 532 nm at the high and low applied voltages, respectively. For the half retardation condition, the signal voltage (3 V_p_ at 1 kHz) is not zero, and the transition of the LC geometry from bent to splayed does not occurr^32^. Thus, as shown in Fig. 4d–f, the fast-switching dynamics can be obtained: the field-on and field-off response times of the polarization switching layer are 270 μs and 180 μs, respectively. The switching dynamics is sufficiently fast such that the presented resolution-enhanced LF imaging system can be applied to real-time image capturing in the case of a moving object. The moving picture showing the switching dynamics of the virtual-moving MLA is presented in Movie S1, wherein the switching results of the periodic focused beam patterns are provided at the low and fast-switching frequencies between two focused states. In Table 1, the detailed specifications of the LF imaging system comprising the virtual-moving MLA are listed.

**Table 1:** Key parameters of the LF imaging system implemented by using the virtual-moving MLA.

	Specification	Value
Main lens	Focal length	50 mm
	*f* -number	*f*/16
	Magnification	1.4×
	Field of view	23°
Virtual-moving MLA	Elemental lens pitch	100 μm
	Focal length	1.6 mm at *λ* = 550 nm
	*f* -number	*f*/16
	Temporal sampling state	Two states
	Operation voltage	30 V_p_
	Total switching time	620 μs
Elemental image array	Resolution of elemental image array captured with single PDMLA	1792 × 1008 pixels
	Resolution of superposed elemental image array captured with virtual-moving MLA	3584 × 2016 pixels
Reconstructed image	Resolution of reconstructed image from single elemental image	128 × 72 pixels
	Resolution of reconstructed image from superposed elemental image	256 × 144 pixels
	Number of directional view	14 (horizontal) × 14 (vertical)
Image sensor	Pixel pitch (single shot)	4.54 μm

## LF Imaging System Utilizing Virtual-Moving MLA

To establish the spatial-resolution enhancement of the LF imaging system without degradation of the angular sampling resolution, we demonstrate a resolution-enhanced LF imaging system using the virtual-moving MLA. The basic concept of LF imaging was developed by Levoy and Hanrahan in 1996 as the capturing of all rays in free space^5^. The reconstructed images through a virtual aperture from the LF images was first demonstrated with a moving camera method by Isaksen in 2000^6^. The basic optical system of the LF imaging consists of a main lens, an MLA, and an image sensor array. The rays from a single point of an object at a desired depth are converged by the main lens on the focal plane of the MLA. The MLA separates these rays according to the direction of each ray and generates elemental images that are recoded by the image sensor array. The number of directional views is determined by the number of pixels recoded by each elemental lens. To increase the number of directional views, the number of pixels covered by each elemental lens is required to be increased. Thus, under a fixed pixel density condition, the pitch of the elemental lens should be increased for obtaining more directional views. However, the spatial resolution of the LF imaging system is determined by the spatial density of the MLA. In the directional view reconstruction, the resolution of the reconstructed image is inherently limited by the number of elemental lenses. The spatially periodic sampling interval is also determined by the pitch of the elemental lens. Based the Nyquist sampling theorem, the upper limit of the spatial resolution (*β*_*nyq*_) is expressed as follows^33^:

2 $${\beta }_{nyq}\approx L/2{P}_{e}$$

in cycles per radian, where *P*_*e*_ is the pitch of the elemental lens, and *L* is the distance between the observation point and the MLA position.

**Figure 5:**
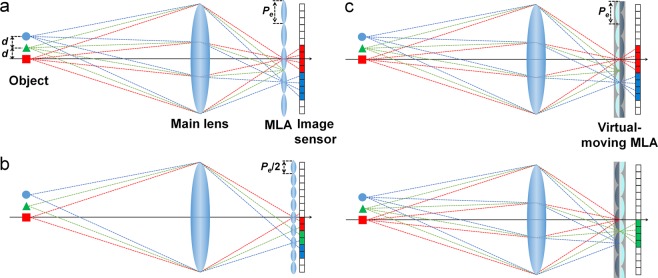
Schematics showing the ray capturing at the image sensor plane in the LF imaging systems: (**a**,**b**) LF image capturing by the conventional single passive MLA with the elemental lens pitch of (**a**) *P*_*e*_ and (**b**) *P*_*e*_/2. (**c**) LF image capturing using the time-multiplexing scheme of the virtual-moving MLA with the elemental lens pitch of *P*_*e*_.

Figure 5 shows the schematics of the LF imaging systems, wherein we assumed that the pixel densities of the image sensors represented in Fig. 5a–c are the same. In Fig. 5a, the sampling interval is twice the distance (*d*) between two object points. While the points indicated by the blue circle and red square are recoded with the angular distribution of the rays, the rays from the green triangle are not recoded as the sampling interval of the LF imaging system is limited by the pitch of the elemental lens. When the pitch of the elemental lens is reduced to half (as shown in Fig. 5b) that of Fig. 5a, the sampling interval becomes *d*. Therefore, the point indicated by the green triangle can be recoded with an increased spatial resolution. However, in this case, the amount of information for the angular ray distribution is decreased by half that shown in Fig. 5a owing to the limited ray sampling rate of the MLA in the LF imaging system. Figure 5c shows the schematic of the time-multiplexing LF imaging system using the virtual-moving MLA, wherein the brightly coloured one of the two PDMLAs represents the PDMLA working as the ray-sampling MLA under one of the incident polarization conditions. The elemental lens pitch of Fig. 5c is the same as that of the MLA presented in Fig. 5a. In addition, the two points indicated by the blue circle and red square can be resolved by the top PDMLA, and the points indicated by the green triangle can also be resolved by the bottom PDMLA without degradation of the angular sampling resolution for all the points as compared to the case in Fig. 5b.

In our experiment, we used a main lens with a 1.4× magnification and a virtual-moving MLA with an elemental lens pitch of 100 μm. On assuming the distance between the observation point and main lens to be approximately 2 m, the sampling interval of the LF imaging system using the single PDMLA is 71.4 μm (=100 μm/1.4), whereas, using the virtual-moving MLA, it can be improved to be 35.7 μm (=100 μm/1.4/2). The details of the optical set-up showing the LF imaging system using the virtual-moving MLA are represented in Fig. S2 (See Supplementary Section 2).

## Synthesis of Elemental Image Array

**Figure 6:**
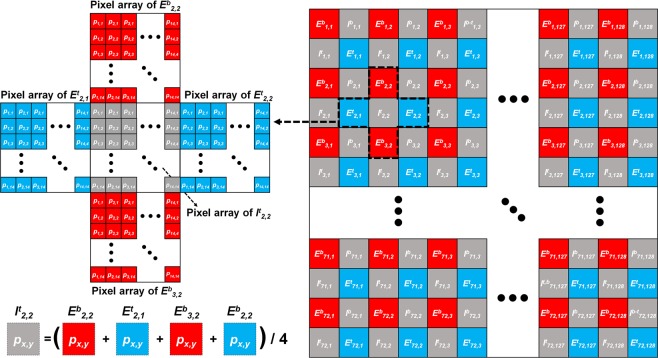
Schematics showing the synthesis of additional elemental image sets (grey-coloured) from two sets of the time-multiplexed elemental images (red- and blue-coloured) captured by the bottom and top PDMLA.

Figure 6 shows the diagram of the synthesis of the elemental image arrays acquired by the resolution-enhanced LF imaging system using the virtual-moving MLA. Using the virtual-moving MLA, two sets of the elemental image arrays are obtained by the top and bottom PDMLA corresponding to the high and low field states applied to the polarization switching layer. As shown in Fig. 6, we denote the [*i*, *j*]-th elemental image captured by the top and bottom PDMLA as *E*^*t*^_*i*,*j*_ and *E*^*b*^_*i*,*j*_, respectively, and the [*x*, *y*]-th pixel for the elemental images as *p*_*x*,*y*_. The elemental image arrays captured by the top and bottom PDMLAs are superimposed and rearranged diagonally, as shown in Fig. 6, wherein there are spatial vacancy positions for the rearranged elemental image sets between *E*^*t*^_*i*,*j*_ and *E*^*b*^_*i*,*j*_ as the virtual-moving MLA is shifted along the diagonal direction only. The spatial vacancy positions of the elemental image sets are filled using linear interpolation. Linear interpolation is a common method used to speculate unknown pixel information^34^. The [*i*, *j*]-th superimposed elemental images at the *E*^*t*^_*i*,*j*_ and *E*^*b*^_*i*,*j*_ columns are denoted as *I*^*t*^_*i*,*j*_ and *I*^*b*^_*i*,*j*_, respectively, and the unknown pixel information is derived using the surrounding pixels information as follows:

3 $${I}_{i,j}^{b}({p}_{x,y})=mean\{{E}_{i,j}^{b}({p}_{x,y}),{E}_{i-1,j}^{t}({p}_{x,y}),{E}_{i,j+1}^{b}({p}_{x,y}),{E}_{i,j}^{t}({p}_{x,y})\}$$

4 $${I}_{i,j}^{b}({p}_{x,y})=mean\{{E}_{i,j}^{b}({p}_{x,y}),{E}_{i,j-1}^{t}({p}_{x,y}),{E}_{i+1,j}^{b}({p}_{x,y}),{E}_{i,j}^{t}({p}_{x,y})\}$$

It should be noted that the superimposed pixel information and the surrounding pixel information in Eqs (3) and (4) have the same [*x*, *y*] index for each elemental image, and *E*^*t*^_*i*,*j*_(*p*_*x*,*y*_), *E*^*b*^_*i*,*j*_(*p*_*x*,*y*_), *I*^*t*^_*i*,*j*_(*p*_*x*,*y*_), and *I*^*b*^_*i*,*j*_(*p*_*x*,*y*_) have the same angular information, but the spatial information is shifted by half the elemental lens pitch. The synthesized elemental image arrays have a spatial resolution that is increased by four times as compared to that of the elemental image arrays obtained with the top or bottom PDMLA only. In this experiment, we used a virtual-moving MLA with 128 × 72 elemental lenses. Each elemental lens recoded the viewing distribution of rays on the 14 × 14-pixel array, and accordingly, the number of directional views were 14 × 14, as listed in Table 1.

## Results and Discussion

To verify the resolution enhancement effect of the LF imaging system using the virtual-moving MLA, the USAF-1951 resolution chart was captured at 150 mm from the main lens. For the reconstructed images, the number of the elemental image sets can be increased just by applying pixel interpolation without increasing the elemental image capturing sets. For the sake of comparison, in this experiment, we compared three cases of the directional view reconstructions from the single set of the elemental image arrays, the linearly interpolated ones by using the single set of the elemental image arrays, and the superimposed and synthesized elemental image array sets based on the two sets of elemental image arrays captured using the virtual-moving MLA.

**Figure 7:**
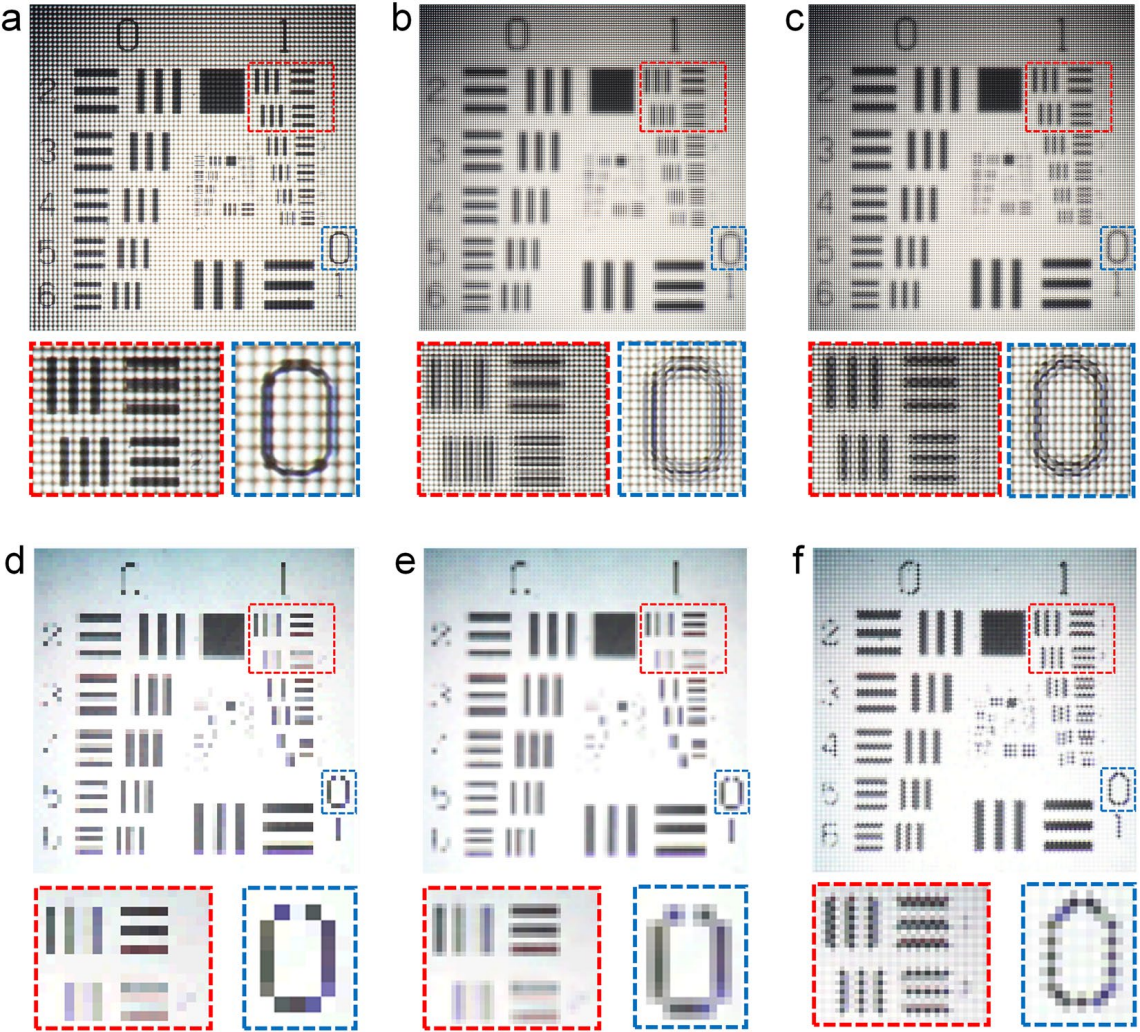
(**a**–**c**) Photographs of the elemental image arrays acquired using the LF imaging systems with the USAF resolution chart as an object. (**a**) Elemental image array captured by the single PDMLA. (**b**) Linearly interpolated elemental image array using (**a**) as an original image. (**c**) Superposed elemental image array based on two sets of the elemental image array captured by the virtual-moving MLA, which is synthesized as shown in the method in Fig. 6. (**d**–**f**) Normal-view images reconstructed from the elemental image arrays are shown in (**b**–**f**).

Figure 7a shows the elemental image arrays captured by the single PDMLA. Figure 7b shows the linearly interpolated elemental image arrays based on the single elemental image arrays, where the number of pixels was increased by four times that of the elemental image arrays shown in Fig. 7a. Figure 7c shows the superimposed and synthesized elemental image arrays based on the time-multiplexed elemental images capturing using the virtual-moving MLA, where the number of image pixels was also increased by four times that of the elemental image arrays shown in Fig. 7a. Figure 7d–f show the reconstructed normal view images from the elemental image arrays corresponding to Fig. 7a–c, respectively.

In Fig. 7, the periods of the two types of line patterns within the red box are 500 μm and 466 μm, respectively, and the character ‘0’ is also within the blue box. In the reconstructed image from the single elemental image array and its enlarged pictures shown in Fig. 7d, there were some reconstructed pixels with information missing, and the intensities of some pattern images were highly non-uniform. For the reconstructed result of the character ‘0’ also, there were image pixels with data missing, and this round-shaped character was depicted in the staircase form. In the case of the single PDMLA, as the ray sampling for the reconstruction was inherently distorted during the recoding process, even after the interpolation process was performed, the distortion of the image reconstruction was not reduced. Instead, the image was shaded and further distorted as shown in Fig. 7e. In the case of the reconstructed image from the superimposed and synthesized elemental image arrays shown in Fig. 7f, the fine patterns of the periodic lines could be resolved well without distortion and blurring, and the round-shaped character ‘0’ was also reconstructed well as compared to Fig. 7d,e.

Figure 7f reconstructed from Fig. 7c shows the fine grid-like patterns, differently with Fig. 7d,e of the reconstructed results with the elemental image arrays captured by the single PDMLA. In the stacked PDMLA structure implemented for the polarization-dependent switching virtual-moving MLA scheme, the optical gap (~20 μm) exists between two PDMLAs. In our experiment, the image plane of the top PDMLA was more ideally set to the image sensor plane rather than the bottom PDMLA. Considering the effective light intensity passing through each pixel aperture of the image sensor, the average brightness level of the elemental image array captured by the top PDMLA was slightly brighter than that by the bottom PDMLA in our experiment. During the superimposed image synthesis (Fig. 6) of the elemental image arrays from two sets of the elemental image arrays captured by the top and bottom PDMLA, the brightness level difference between two sets of the elemental images causes periodic brightness variation and this causes the grid-like patterns in the directional view reconstruction image as shown in Fig. 7f. To resolve this issue, a smaller gap between two lens planes or a larger pixel aperture condition of an image sensor is more desirable, an LF imaging system based on a lens condition with a smaller field of view is more suitable, and a stacked PDMLA with two different focal lengths can be implemented. More simply, the reconstructed image issue of the grid-like patterns can be effectively mitigated by calibrating the brightness levels between two sets of the elemental image arrays before the superimposed image synthesis process, as shown in Fig. S3 (see Supplementary Information S3).

**Figure 8:**
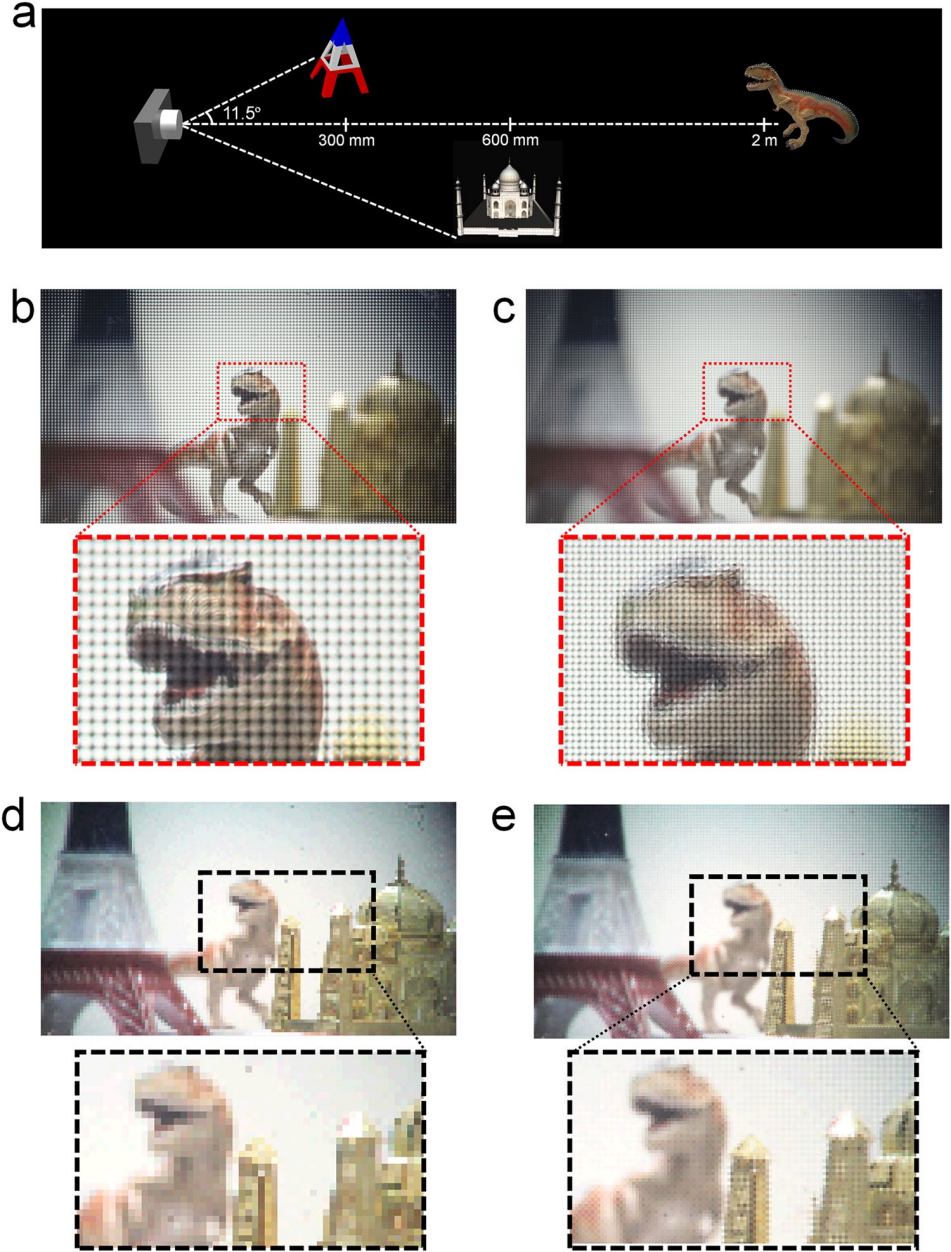
(**a**) Schematics of arrangement of the 3D real objects used in the LF imaging experiment. (**b**) Photograph of the elemental image arrays captured by the single PDMLA, and its enlarged picture. (**c**) Photograph of the elemental image arrays synthesized using two sets of the elemental image arrays with the time-multiplexed virtual-moving MLA, and its enlarged picture. (**d**,**e**) Reconstructed images for the normal view condition obtained using the elemental image sets of (**b**,**c**), respectively. For the reconstructed directional-view moving pictures showing the conventional and resolution-enhanced images, see Movie S2.

To demonstrate and compare the LF reconstruction results of the directional view images and the depth-slice refocused images, three individual volume objects were captured as shown in Fig. 8a. The three objects were located at 300 mm, 600 mm, and 2 m from the main lens within 23° of the viewing window. The image plane of the main lens was set to the furthest object of the dinosaur. Figure 8b,c show the elemental image arrays captured by the single PDMLA and the superimposed elemental image array based on the elemental images captured by the virtual-moving MLA, respectively, where the number of the elemental images for Fig. 8b,c were 128 × 72 and 256 × 144, respectively. For both conditions, each elemental lens recoded the viewing distribution of the rays from the objects to the 14 × 14-pixel array of the image sensor. In Fig. S4 (see Supplementary Information S4), we presented the elemental image arrays captured by the single PDMLA (the bottom or top PDMLA) and the superimposed and synthesized elemental image arrays captured by the virtual-moving MLA. From each captured elemental image array shown in Fig. 8b,c, the LF image reconstruction was performed for the directional view images. Figure 8d,e show the reconstructed normal view images from the single elemental image arrays and the superimposed elemental image arrays. The number of pixels of the reconstructed view image was increased from 128 × 72 in Fig. 8d to 256 × 144 in Fig. 8e. As shown in the enlarged pictures of Fig. 8d,e, the reconstructed image obtained using the proposed LF imaging system with the virtual-moving MLA presents the significantly enhanced spatial resolution at the same angular sampling resolution. The other sets for the different view directions are presented in Fig. S5 (see Supplementary Information S5) and Movie S2.

**Figure 9:**
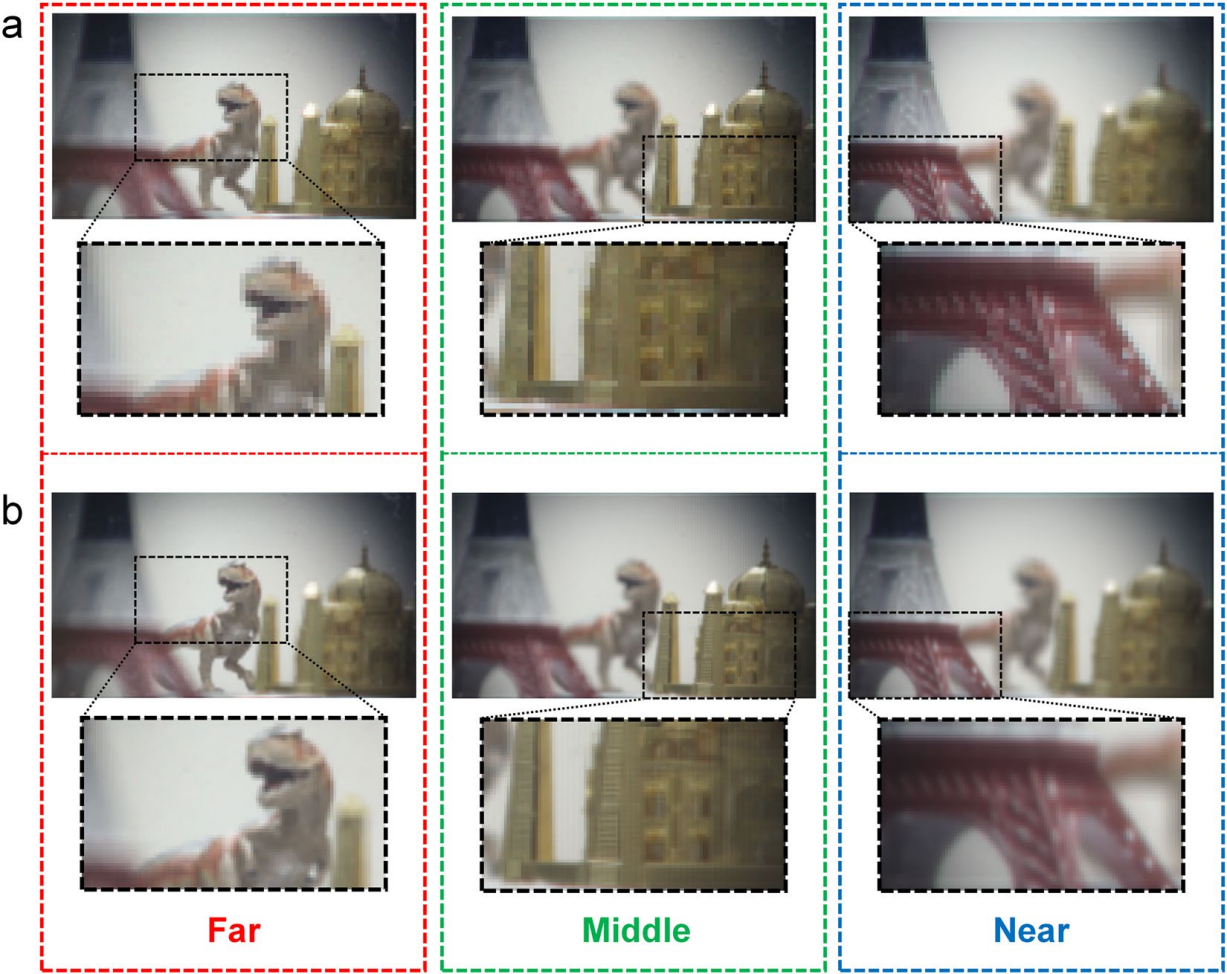
Depth-slice images reconstructed for the far (2 m), middle (600 mm), and near (300 mm) planes. (**a**) Reconstructed images from the single elemental image sets shown in Fig. 8b and captured by the single PDMLA. (**b**) Reconstructed images from the superimposed elemental image sets shown in Fig. 8c and captured by the virtual-moving MLA. For the reconstructed depth-refocused moving pictures showing the conventional and resolution-enhanced images, see Movie S3.

Figure 9 shows the depth-slicing refocused images reconstructed from the elemental image arrays; these images were obtained using the computational integral imaging reconstruction method (CIIR)^26,35,36^, wherein the images focused on the desired depth plane were reconstructed for the distances of 2 m, 600 mm, and 300 mm. Figure 9a shows the reconstructed depth-slice images from the elemental image arrays captured by the single PDMLA, and Fig. 9b shows the reconstructed depth-slice images from the superimposed elemental image arrays captured by the time-multiplexed virtual-moving MLA. Differently with the reconstructed results for the directional view images, the reconstructed depth-slice images from the superimposed elemental image arrays do not exhibit the grid-like image patterns, as shown in Fig. 9b, although the brightness level calibration on two sets of original elemental image arrays are not performed in this case. For the depth-slice image reconstruction, the CIIR method utilizes average values from multiple sets of non-periodically sampled pixels corresponding to the desired depth information and the periodic grid-like pattern is disappeared. The image resolutions of Fig. 9a,b were 128 × 72 and 256 × 144, respectively. It is also apparent that the reconstructed depth-slice images from the proposed LF imaging system using the virtual-moving MLA exhibited a significantly improved spatial resolution at the same angular resolution.

## Conclusions

We demonstrated a resolution-enhanced LF imaging system by introducing an electrically controllable fast-switching virtual-moving MLA. The periodic ray sampling position of the virtual-moving MLA could be spatially shifted with a very short switching time by controlling the incident polarization state of the polarization switching layer. Our switchable lens scheme does not comprise any physical moving part and is free from the complex LC molecular dynamics existing in the LC-based switchable lens. The presented virtual-moving MLA could exhibit a fill factor of nearly 100% with a well-aligned LCP texture. Using the fast-switching virtual-moving MLA, we demonstrated the resolution-enhanced LF imaging system. In this systems, two sets of the elemental image arrays were captured time-sequentially at a high image-capturing rate, where the field-on and field-off response times of the virtual-moving MLA were 270 μs and 180 μs, respectively. From the two captured sets of the elemental image arrays, the four-times resolution-enhanced images of the directional-view and depth-slice images could be reconstructed without a decrease in the angular sampling resolution as compared to that of the conventional LF imaging system using a passive MLA. The presented fast-switching virtual-moving MLA scheme and its compact LF imaging system shows potential for several real-time 3D imaging applications such as 3D cameras, mobile phone cameras for 3D augmented reality, robot vision systems^37^, smart-flying drone applications^38^, and 3D microscopes for live cell imaging^39^. Especially, the presented LF imaging scheme is simply achievable by implementing our compact and lightweight active switching optical units. Our approach can provide an effective way for miniaturizing conventional endoscope bio-imaging systems which need mechanical zooming or actuation units for searching or imaging of affected areas^40,41^.

## Supplementary information

Movie S1

Movie S2

Movie S3

Supplementary-Information
