# Supplementary material for: Fast-switching laterally virtual-moving microlens array for enhancing spatial resolution in light-field imaging system without degradation of angular sampling resolution
Source: Sci Rep. 2019 Aug 5;9:11297. doi: 10.1038/s41598-019-47819-9 (PMC6683179; doi:10.1038/s41598-019-47819-9)
Supplement: Supplementary file 4 — Supplementary-Information [file 41598_2019_47819_MOESM4_ESM.pdf]

Fast-switching laterally virtual-moving microlens array for enhancing spatial resolution in light-field imaging system without degradation of angular sampling resolution

Min-Kyu Park[1], Heewon Park[1], Kyung-Il Joo[1], Tae-Hyun Lee[1], Ki-Chul Kwon[2], Munkh-Uchral Erdenebat[2], Young-Tae Lim[2], Nam Kim[**,2], and Hak-Rin Kim[*,1]

[1] School of Electronics Engineering, Kyungpook National University, 80 Daehak-ro, Buk-gu, Daegu 41566, South Korea.

[2] School of Information and Communication Engineering, Chungbuk National University, 1 Chungdae-ro, Seowon-gu, Cheongju, Chungbuk 28644, South Korea.

[*]Corresponding author: rineey@knu.ac.kr

[**]Co-corresponding author: namkim@chungbuk.ac.kr

Supplementary Information

Supplementary Section 1. Evaluation of chromatic aberration for polarization-dependent microlens array (PDMLA)

To evaluate the wavelength-dependent focusing and switching properties of the virtual-moving microlens array (MLA) operation, we measured the ordinary (n_o) and extraordinary (n_e) refractive indices of the liquid crystalline polymer (LCP) layer and the refractive index (n_p) of the isotropic polymer layer at the red (650 nm), green (550 nm), blue (450 nm) wavelength conditions by using Fabry-Pérot interferometric method[S1]. The resonant peak wavelengths are determined by the optical path length of the inner media within the Fabry-Pérot cavity and the wavelength-dependent refractive index of the inner media can be obtained from the multiple resonant peaks. To obtain multiple resonant peaks more precisely with the enhanced fringe visibility of the transmission spectra, thin aluminuim (Al) layer was deposited with 20 nm thickness onto the inner surfaces of two glass substrates sandwitched for the Fabry-Pérot cavity. For measuring the refractive indices (n_o and n_e) of the

unidirectionally ordered LCP layer, a rubbed polyvinyl alcohol (PVA) layer was formed onto the Al layer as a liquid crystal alignment surface. For measuring the refractive index (n_p) of the isotropic polymer layer, the PVA layer coating was not performed on the Al surface. The gap of the Fabry-Pérot cavity was maintained by silica spacers of 20 μm thickness. Two types of the Fabry-Pérot cells were prepared by capillary filling of the LCP or isotropic polymer materials into the Fabry-Pérot cavity. The inner layers of two Fabry-Pérot cells were photo-polymerized by UV irradiation. In case of the LCP-filled Fabry-Pérot cells, the cavity medium has an optically uniaxial property due to the molecular ordered state of the LCP layer and its transmission spectra show the polarization-dependent resonant peak behaviours[S1]. For two Fabry-Pérot cells, the transmission spectra were measured by using a UV-vis spectrophotometer (JASCO V-650, JASCO). For the Fabry-Pérot cell with the isotropic polymer as the inner layer, an unpolarized beam was irradiated as the probe beam. For the Fabry-Pérot cell with the LCP layer, a linearly polarized beam was irradiated with changing its polarization state orthogonally.

The measured values of the refractive indices are n_o=1.5592, n_e=1.7511, and n_p=1.5652 at λ=450 nm (blue), and n_o=1.5136, n_e=1.6794, and n_p=1.5111 at λ=550 nm (green), and n_o=1.4952, n_e=1.6508, and n_p=1.4892 at λ=650 nm (red). Using the curve fitting with the Cauchy equation[S1,S2,S3], the wavelength-dependent dispersion curves were obtained for n_o, n_e, and n_p, as shown in Fig. S1a. The dispersion equations are as follows.

$$n_o(\lambda) = 1.47400 + \frac{0.00129}{(\lambda/\mu m)^2} + \frac{0.00323}{(\lambda/\mu m)^4}, \quad \text{(S1)}$$

$$n_e(\lambda) = 1.62000 + \frac{0.00057}{(\lambda/\mu m)^2} + \frac{0.00526}{(\lambda/\mu m)^4}, \quad \text{(S2)}$$

$$n_p(\lambda) = 1.46400 + \frac{0.00160}{(\lambda/\mu m)^2} + \frac{0.00382}{(\lambda/\mu m)^4}. \quad \text{(S3)}$$

By using these dispersion curves, the dispersion properties of the (n_e - n_p) and (n_o - n_p) values were evaluated as shown in Fig. S1b. The wavelength-dependent refractive index values of n_o and n_p show the highly similar dispersion properties with satisfying the index matching condition between the LCP and isotropic polymer layers over the whole visible range. The average value ($<|n_o - n_p|>$) of the refractive index difference between two layers over the visible range (400 nm ~ 700 nm) was

$< |n_o - n_p| >$ = 0.005615. This means that the index matching condition between n_o of the planar-convex LCP layer and n_p of the planar-concave isotropic polymer layer can be well preserved over the visible range although both of n_o and n_p values have the unavoidable material dispersions, which is required to avoid the image crosstalk problem between two sets of the elemental image arrays in our virtual moving MLA scheme.

Compared with the (n_o - n_p) values, the (n_e - n_p) values show a higher wavelength dependency as shown in Fig. S1b because the liquid crystal (LC) materials have a larger dispersion properties for n_e than n_o due to their uniaxial molecular structure [S2,S3]. This results in the chromatic aberration properties in a focused state like several types of LC-based lens[S4]. We simulated the wavelength-dependent focusing characteristics of the polarization-dependent microlens array (PDMLA) by utilizing the optical modeling software of Advanced System Analysis Program (ASAP™, Breault Research Organization, Inc.) based on the material dispersion data shown in Fig. S1a. The focal length conditions of the PDMLA were 1.65 mm, 1.60 mm, and 1.47 mm for the red (650 nm), green (550 nm), and blue (450 nm) wavelengths, respectively, under the extraordinary ray incidence condition, as shown in Fig. S1c.

Considering these chromatic aberration properties of the PDMLA, a lens design for the PDMLA with a relatively large *f*-number (*f*/16) was introduced in our light-field (LF) imaging system to provide an image acquisition condition with a sufficiently large depth of field. So, the chromatic aberration effect of the PDMLA could be mitigated in our LF imaging results as shown in Figs. 8 and 9. The wavelength dependency of the image-resolving capability of the PDMLA was characterized by measuring the modulation transfer function (MTF) at the red (650 nm), green (550 nm), blue (450 nm) wavelengths, where the image plane was fixed at the depth plane optimized for the green wavelength condition. From the results (Fig. S1d), we can see that the MTF values for red and blue wavelengths were not significantly degraded from the MTF values for green wavelength.

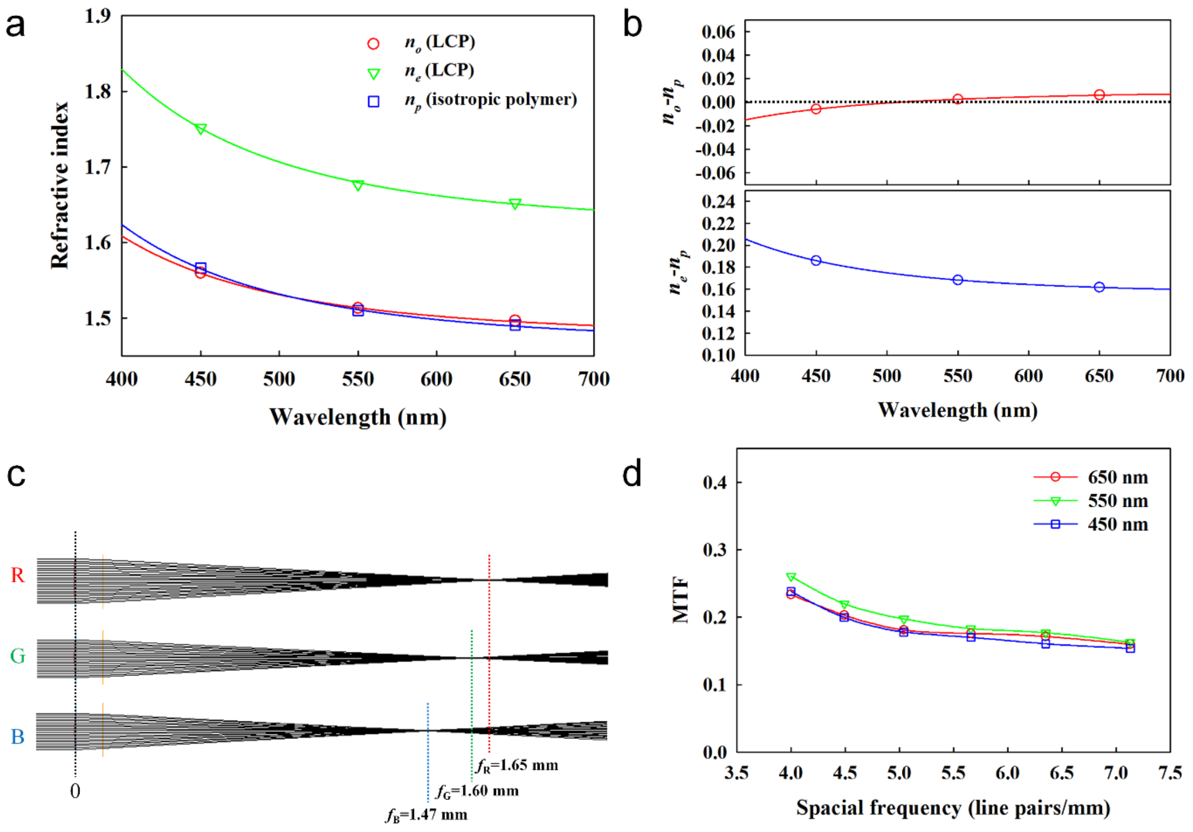

Figure S1. (**a**) Wavelength-dependent refractive indices for the LCP (n_e and n_o: extraordinary and ordinary refractive index, respectively) and the isotropic polymer (n_p), where the symbols are the measured results and the dispersion curves are the fitting results using the Cauchy equation. (**b**) Wavelength-dependent amount of the refractive index difference (n_e - n_p and n_o - n_p) between the LCP and the isotropic polymer, obtained from Fig. **S1a**. (**c**) Ray simulation results for characterizing the wavelength-dependent focusing behaviour of the PDMLA. (**d**) MTF curves of the PDMLA, characterized by using the 1951 USAF resolution test chart at red, green, blue wavelength conditions.

Supplementary Section 2. Optical set-up of the resolution-enhanced light-field (LF) imaging system using the fast-switching virtual-moving microlens array (MLA)

Figure S2 shows the optical set-up of the resolution-enhanced LF imaging system using the virtual-moving MLA. We placed the virtual-moving MLA on the focal plane of the main lens (AF Nikko f/1.4D, Nikon Ltd.) to image objects using the virtual-moving MLA. To obtain a fast-switching operation with the polarization-dependent virtual-moving MLA, a polarizer and a polarization switching layer with an optically compensated bend (OCB) LC mode are installed in front of the main lens. The transmission axis of the polarizer is parallel with the y-axis, and the rubbing direction of the OCB LC cell is 45° with respect to the x–y axes. As the image sensor, we used a camera (EOS 80D, Canon Inc.) with a relay lens (EF 100mm f/2.8L Macro IS USM, Canon Inc.) used to recode the elemental image arrays captured by the virtual-moving MLA. To maximize the fill factor of each elemental image set and to reduce image-overlapping problem between the adjacent elemental images, we designed the virtual-moving MLA to have an f-number (f/16) that is the same as that of the main lens. The elemental image sets were captured by the image sensor through the relay lens (f/2.8), and the shutter speed of the camera was 1/50 s.

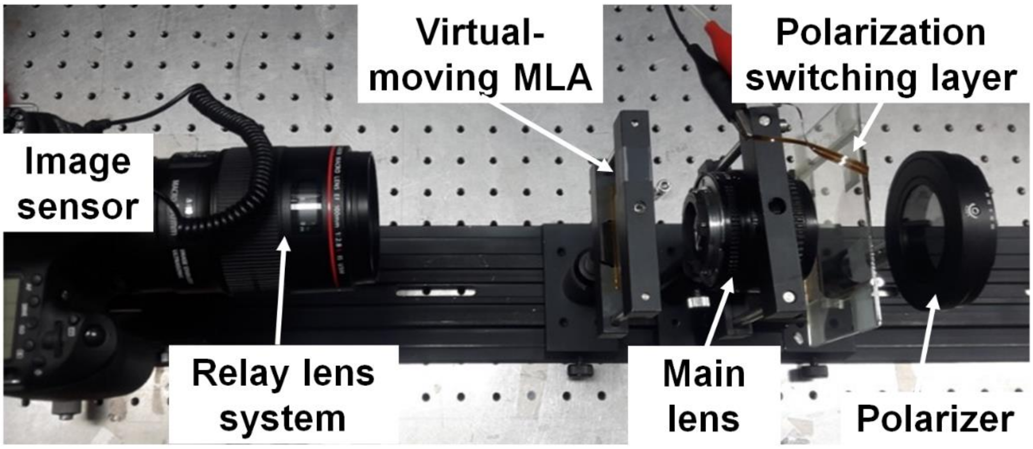

Figure S2. Optical set-up of the resolution-enhanced LF imaging system using the time-multiplexing scheme of the fast-switching virtual-moving MLA.

Supplementary Section 3. Elemental image arrays and reconstructed images

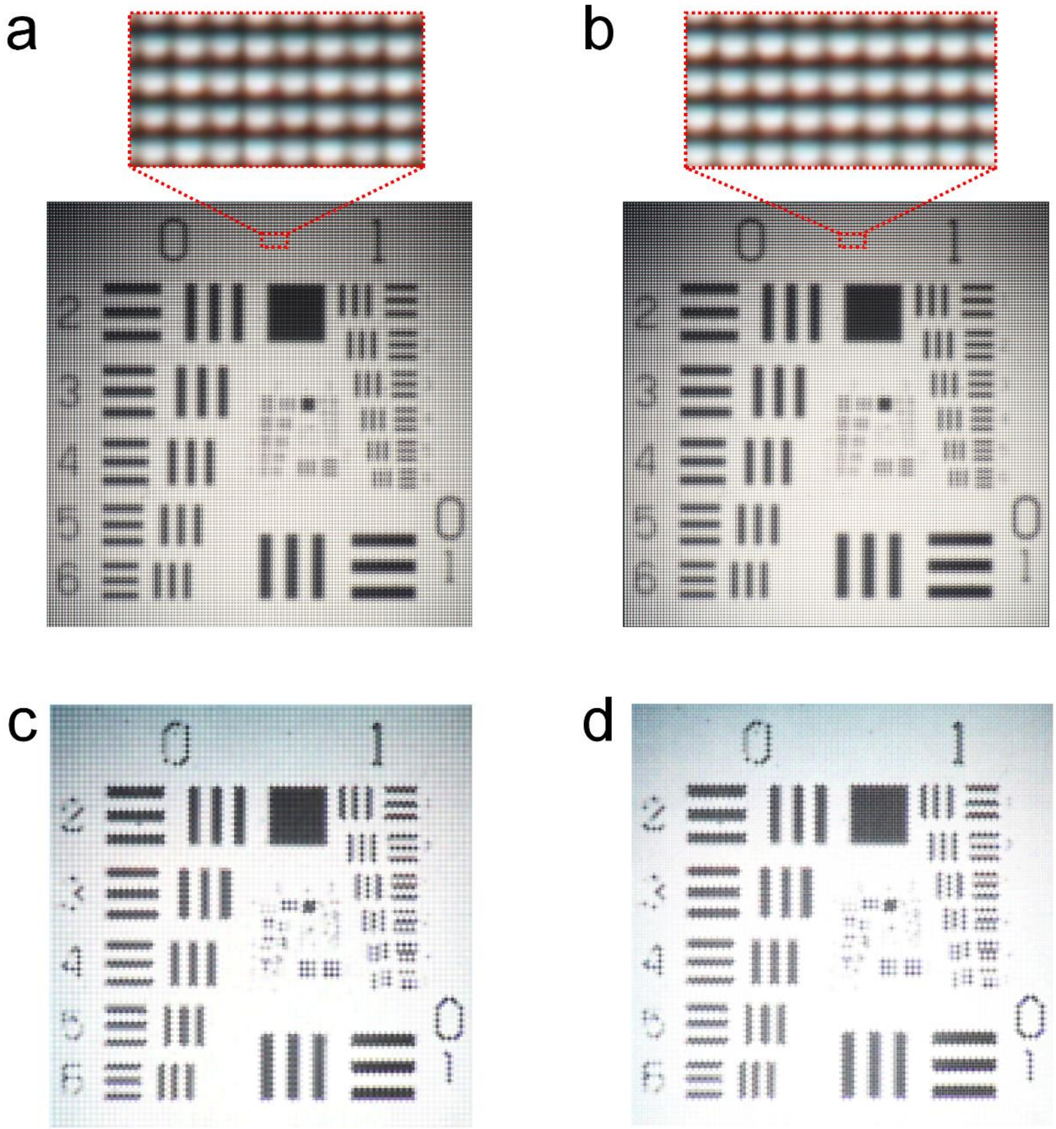

Figure S3. (**a** and **b**) Photographs of the superposed elemental image array based on two sets of the elemental image arrays captured by the virtual-moving MLA: (**a**) without the brightness calibration and (**b**) with the brightness calibration between two elemental image sets. (**c** and **d**) Normal-view images reconstructed from the elemental image arrays of (**c**) Fig. **S2a** and (**d**) Fig. **S2b**.

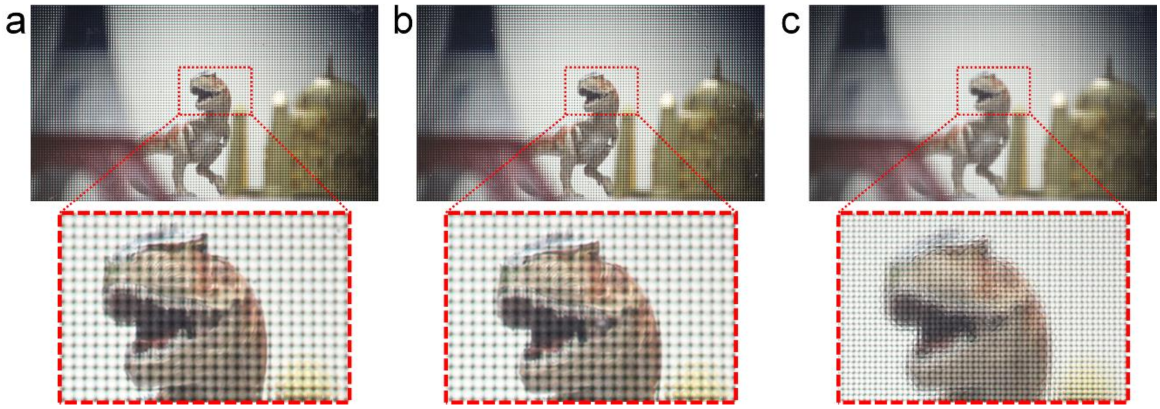

Figure S4. Photographs of the elemental image arrays captured from (**a**) the bottom polarization-dependent MLA (PDMLA) and (**b**) top PDMLA, and (**c**) the synthesized elemental image arrays based on two sets of the elemental image arrays captured by the virtual-moving MLA.

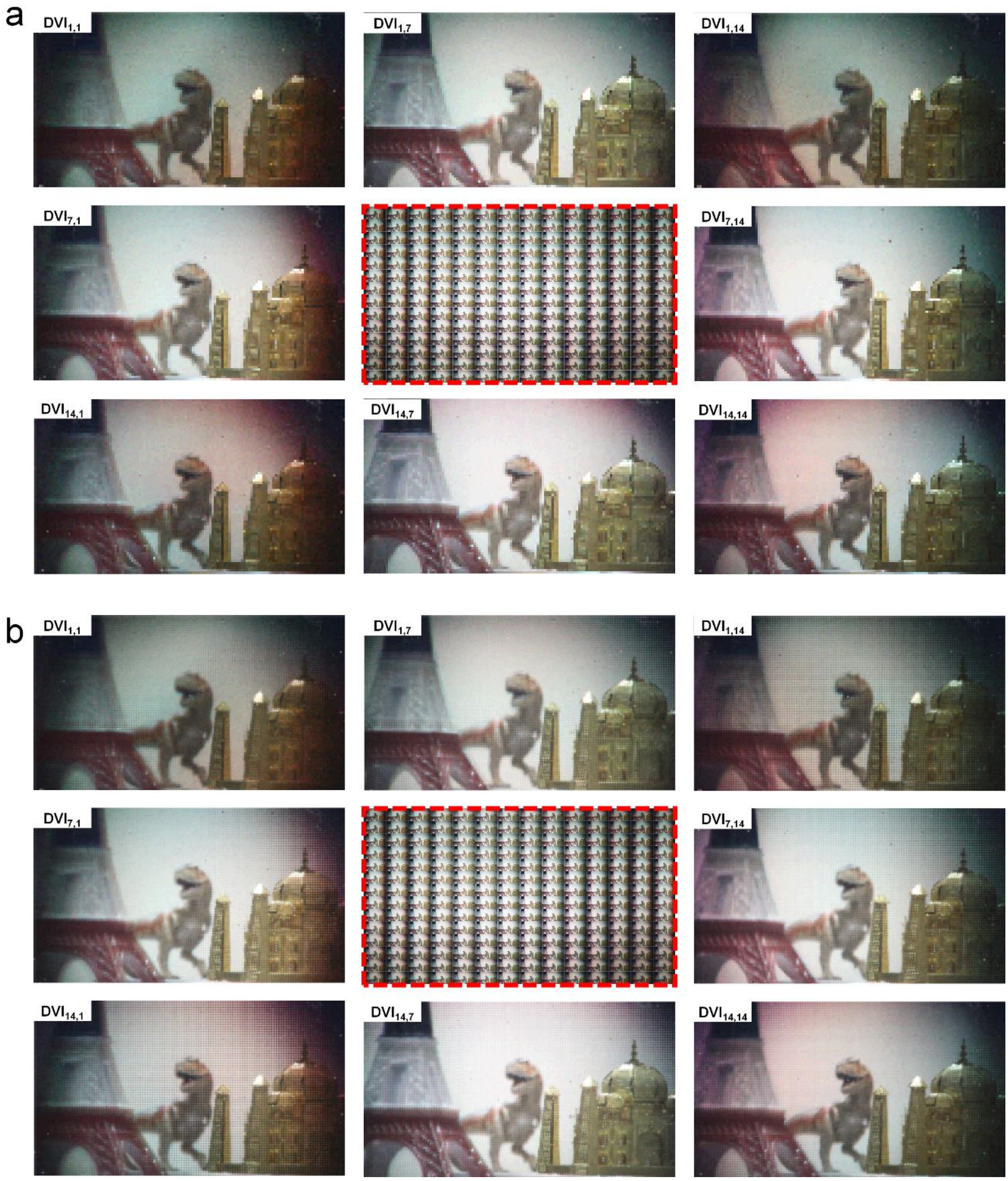

Figure S5. Directional-view images reconstructed from the elemental image arrays. The photographs in the red boxes are all sets of the directional-view images, and $DVI_{x,y}$ represents the reconstructed image with $p_{x,y}$. (a) Directional-view images from the elemental image array captured from the single PDMLA. (b) Directional-view images from the superimposed and synthesized elemental image array based on two sets of the elemental image arrays captured by the virtual-moving MLA.

References

S1. Kim, H.-R., Jang, E., Im, J. & Lee, S-D. Precise Determination of Thermal Effect on the Optical Properties of Liquid Crystal Devices Using the Fabry–Perot Interferometry. *Japanese Journal of Applied Physics* **44**, 294–297 (2005).

S2. Wu, S.-T. Birefringence dispersions of liquid crystals. *Physical Review A* **33**, 1270–1274 (1986).

S3. Li, J. & Wu, S.-T. Extended Cauchy equations for the refractive indices of liquid crystals. *Journal of Applied Physics* **95**, 896–901 (2004).

S4. Ren, H., Xianyu, H., Xu, S. & Wu, S.-T. Adaptive dielectric liquid lens. *Optics Express* **16**, 14954–14960 (2008).
